# AREsite2: an enhanced database for the comprehensive investigation of AU/GU/U-rich elements

**DOI:** 10.1093/nar/gkv1238

**Published:** 2015-11-23

**Authors:** Jörg Fallmann, Vitaly Sedlyarov, Andrea Tanzer, Pavel Kovarik, Ivo L. Hofacker

**Affiliations:** 1 Institute for Theoretical Chemistry, University of Vienna, Währingerstraße 17/3, A-1090 Vienna, Austria; 2 Max F. Perutz Laboratories, University of Vienna, Dr. Bohr-Gasse 9, A-1030 Vienna, Austria; 3 Research Group Bioinformatics and Computational Biology, Faculty of Computer Science, University of Vienna, Währingerstraße 29, A-1090 Vienna, Austria; 4 Center for non-coding RNA in Technology and Health, University of Copenhagen, Grønnegårdsvej 3, DK-1870 Frederiksberg C, Denmark

## Abstract

AREsite2 represents an update for AREsite, an on-line resource for the investigation of AU-rich elements (ARE) in human and mouse mRNA 3′UTR sequences. The new updated and enhanced version allows detailed investigation of AU, GU and U-rich elements (ARE, GRE, URE) in the transcriptome of *Homo sapiens*, *Mus musculus*, *Danio rerio*, *Caenorhabditis elegans* and *Drosophila melanogaster*. It contains information on genomic location, genic context, RNA secondary structure context and conservation of annotated motifs. Improvements include annotation of motifs not only in 3′UTRs but in the whole gene body including introns, additional genomes, and locally stable secondary structures from genome wide scans. Furthermore, we include data from CLIP-Seq experiments in order to highlight motifs with validated protein interaction. Additionally, we provide a REST interface for experienced users to interact with the database in a semi-automated manner. The database is publicly available at: http://rna.tbi.univie.ac.at/AREsite

## INTRODUCTION

AU-rich elements (AREs) and GU- or U- rich elements (G/UREs) are sequence motifs found in many coding and non-coding RNAs. Upon interaction with RNA-binding proteins (RBPs) they can influence the half-life of RNA molecules. This interaction can induce RNA stabilization or destabilization, mediated by mechanisms that depend on the RBP and the genic motif context, but are otherwise not fully understood. The most prominent example is an important gene expression regulating mechanism known as AU-rich element mediated decay (AMD) (1).

However, AMD is not the only RNA stability regulating process that depends on successful RNA-RBP interaction. RBPs interact e.g. with GU-rich elements (GRE), as well as U-rich elements (UREs) that have also been shown to modulate mRNA half-life (2–5).

So far, mostly protein coding genes have been shown to be regulated by these mechanisms and only 3′UTR binding was shown to regulate mRNA half-life (6). Only recently CLIP-Seq (7) was introduced as a new method to identify RBP binding sites in a high-throughput manner. These CLIP-Seq experiments, identified many novel binding sites for RNA-binding proteins (RBP) involved in RNA regulation (see e.g. (4,8–10), etc.), showing significant binding of RBPs in genic regions like introns or 5′UTRs, with unknown regulatory function. Furthermore, experiments show that binding sites often contain only partial matches with previously annotated motifs, such that a more relaxed view of motif preferences has become necessary. Therefore, the research community faces novel challenges regarding the investigation of RNA-RBP interplay beyond current paradigms. *In silico* methods play an important role in the identification of (novel) binding sites and the prediction of their regulatory role. Established databases like ARED (11), GRED (5), AURA (12) or the old AREsite (13) provide the user with information on motif location, accessibility and more, but are not designed to cope with more recent findings and high-throughput requests. On the one hand AREsite focuses solely on 3′UTRs of protein coding genes, while ARED and GRED are very restricted regarding motifs. More than 40 citations and 45 000 visitors, underline the need for new comprehensive bioinformatical resources in this research area, made publicly available now with AREsite2 at http://rna.tbi.univie.ac.at/AREsite

## IMPROVEMENTS

AREsite2 accounts for recent developments by extending its analysis approach to the whole gene body, instead of restricting it to 3′UTRs or introns. The choice of region of interest remains with the user.

Furthermore, by applying more relaxed motif pattern definitions than e.g. ARED for annotation, we aim at a high coverage of experimentally validated and candidate binding sites relevant for interaction, dynamics and mechanisms of RNA-RBP interaction.

Experimentally validated binding sites are a solid basis for the detailed investigation of RNA elements that interact with proteins. To improve our annotation of motifs in this new release, we include binding sites from CLIPdb (14) pre-processed datasets for the prominent RBPs ELAVL1 (HuR), Zfp36 (TTP) and HNRNPD1 (Auf1) where available. Additionally, we will integrate new binding sites from experimental data when they become available, as we did for example with data from Mukherjee *et.al*. (10).

AREsite was to our knowledge the first database including the local structuredness of ARE motif sites in terms of opening energies and accessibilities. As RNA secondary structure proves important for successful RNA-RBP interactions, we integrated RNAplfold (15) derived accessibilities also in this new release. To further improve this feature, AREsite2 incorporates stable secondary structures in overlap with annotated motifs from genome wide scans with RNALfoldZ (16,17). Z-score filtered locally stable RNA secondary structures were predicted for all included genomes and visualization is embedded using forna (18).

The comprehensive manual literature search of version 1 was automated by interaction with PUBMED via the ENTREZ API.

Information retrieval for the experienced user with the need for semi-automatic requests is now possible via a REST interface.

Table 1 provides a short comparison of supported features and changes between AREsite in versions 1 and 2.

**Table 1.** Summary of features in AREsite and AREsite2, respectively

	AREsite	AREsite2
**Genic features**		
3′UTRs	Yes	Yes
5′UTRs		Yes
CDS		Yes
Introns		Yes
mRNAs	Yes	Yes
Non-coding RNAs		Yes
		
**Species**		
*H. sapiens*	Yes	Yes
*M. musculus*	Yes	Yes
*D. rerio*		Yes
*D. melanogaster*		Yes
*C. elegans*		Yes
		
**Motif features**		
AREs	Yes	Yes
UREs/GREs		Yes
Motif accessibility	Yes	Yes
Secondary structures in overlap		Yes
Conservation information	Yes	Yes
Result download	Yes	Yes
Database dump		Yes
Related literature	Yes	Yes
REST interface		Yes
Experimental evidence		Yes

Table 1 highlights differences between AREsite and AREsite2.

Furthermore the backend was changed to a relational database system, allowing dumps of the whole database to be retrieved by the user and easing maintenance and updates of the database with new experimental results, annotations and species.

### Genomes and annotation

Following genomes were used for annotation of motifs and secondary structure prediction

*H. sapiens*, hg38: GRCh38.p2 (Genome Reference Consortium Human Build 38), INSDC Assembly GCA_000001405.17, December 2013

*M. musculus*, mm10: GRCm38.p3 (Genome Reference Consortium Mouse Reference 38), INSDC Assembly GCA_000001635.5, January 2012

*D. rerio*, zv9: Zv9 (The Danio rerio Sequencing Project assembly Zv9), INSDC Assembly GCA_000002035.2, April 2010

*D. melanogaster*, BDGP6: Berkeley Drosophila Genome Project (BDGP) assembly release 6, July 2014

*C. elegans*, WBcel235: WS245 release of WormBase (which includes the WBcel235 version of the *C. elegans* reference genome) INSDC Assembly GCA_000002985.3, December 2012

Gene and transcript annotation for all genomes was retrieved from ENSEMBL (19) version 79 via their ENSEMBL perl API. AREsite2 contains A/G/URE annotations for ∼60 000 genes in *H. sapiens*, ∼43 000 genes in *M. musculus*, ∼35 000 genes in *D. melanogaster*, ∼17 000 genes in *D. rerio* and ∼47 000 in *C. elegans*, multiplying the information content compared to version 1.

### Motifs

While the previous release of AREsite includes only motifs ranging from the ARE core motif ATTTA to its extended 13-mer version WWWWATTTAWWWW, recent experiments (4,8–10) have shown that this is not enough to cover the broad variation of RBP target motifs. With this new release we cover a far broader spectrum of AU/G/U-rich motifs. Together with the fact that we do no longer focus on 3′UTR regions only, but include the whole gene body, as well as non-protein coding genes, the database has undergone a significant increase in size. However, this vast increase in annotated motifs also means that more motifs without (known) regulatory function are now included in the database. To cope with that and improve the gain of knowledge, we decided to integrate experimentally validated target sites of TTP, HuR and Auf1, being the most prominent RBPs involved in mRNA halflife regulation, and highlight them for the end user. To that purpose we used Bedtools (20) and extracted intersections of annotated motifs and experimental results derived from CLIPdb or directly from source (e.g. (10)). Motifs in overlap with CLIP signal are color coded in the output page (TTP red, Auf1 blue, HuR green, multiple bright red, no overlap gray). Motifs annotated for the gene of interest are collected in a sortable table that can be downloaded in bed, xlsx or pdf format, if overlaps with experimental data was detected, links to the corresponding dataset are provided.

### Structural context

Secondary structure of an RNA molecule influences the binding probability of RBPs. Most ABPs are for example known to prefer single-stranded RNA molecules for interaction. Thus, we applied RNAplfold to predict the probabilities of being unpaired for stretches ±20nt around annotated motifs. As in version 1 of AREsite results of this analysis are rendered as downloadable SVGs and help to check the accessibility of motifs of interest for RBPs. Furthermore, we integrated the results of genome wide RNALfoldZ screens for locally stable RNA secondary structures. Overlaps of annotated motifs with *Z*-score filtered stable structures were predicted for all included genomes and are part of the output. If overlaps are found, the user can investigate the structure via a linkout to forna(18).

### Conservation

Information on the conservation of the region of interest is provided at two stages. Once for the gene of interest, where we plot ENSEMBL (19) GERP (21) conservation scores for the whole gene body where available. Additionally, we provide multiple sequence alignments, retrieved from ENSEMBL genomic alignments where available, for annotated motifs to visualize conservation on a per motif scale.

### Literature

The ENTREZ API makes it possible to programmatically fetch publications from PUBMED for a given search string. This allows us to retrieve publications for each gene of interest in context of A/U/GRE motifs and binding proteins respectively. However, the main advantages of automatically retrieved publications is that we stay up-to-date with PUBMED. For convenience and transparency the user can follow the link to PubMed, which contains the used search string, to manually query from PUBMED.

### Statistics

At http://rna.tbi.univie.ac.at/AREsite/statistics, we provide an interface for the user to request the number of genes containing at least one motif of interest in their gene body. The generated bar plot illustrates how many genes contain the selected motif in either intronic or exonic parts of 3′UTR, 5′UTR, CDS and total.

## RESULTS

**Figure 1.**
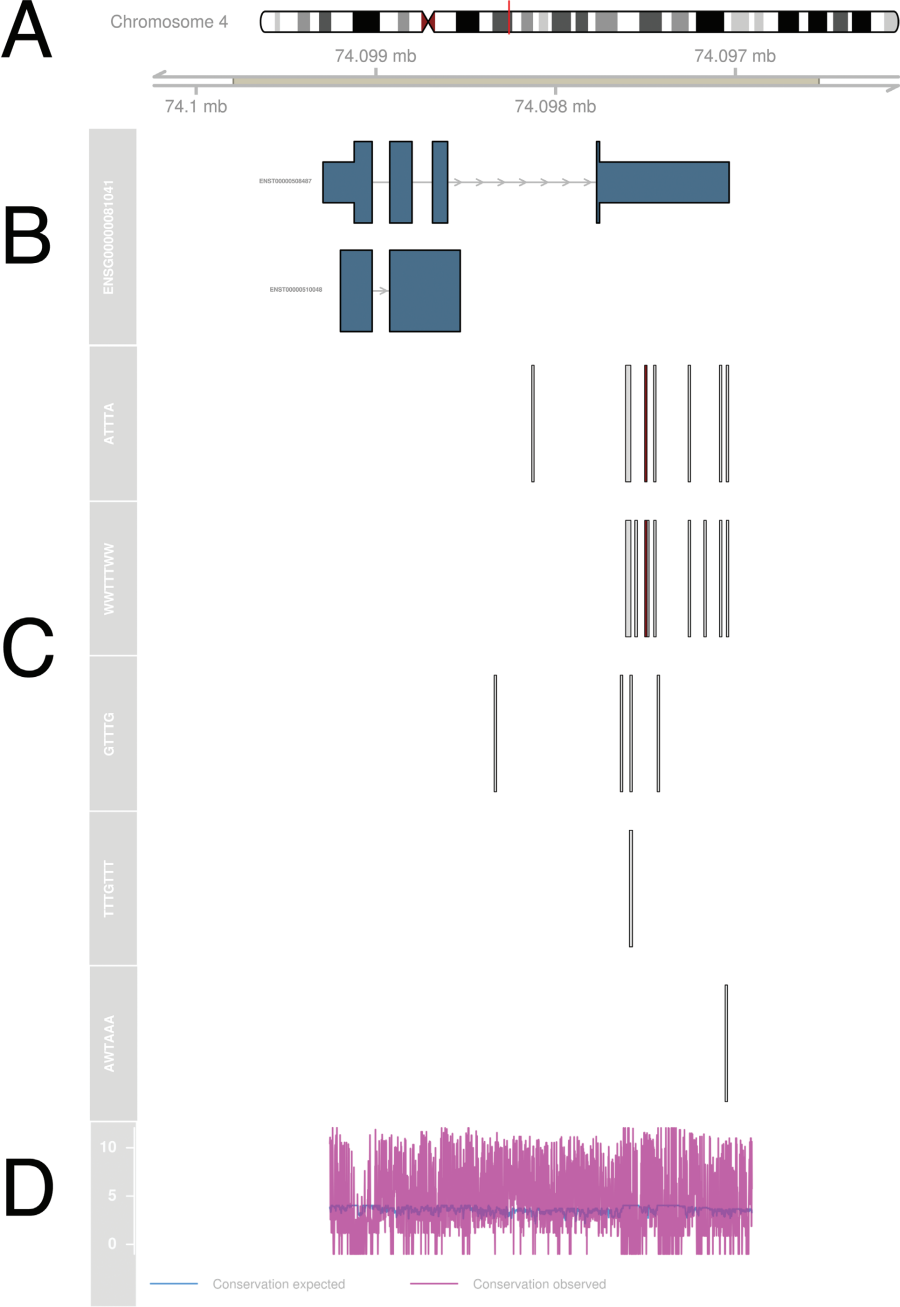
(**A**) Idiogram of hg38 chromosome 4, the location of Cxcl2 is highlighted (**B**) ENSEMBL (19) annotated known transcripts for Cxcl2. Exons are shown as boxes and introns as lines. The genome axis plot above indicates the orientation of the gene and its genomic location. (**C**) Together with Figure 1B, this plot highlights the genic location of annotated motifs and shows overlaps with experimental data in color code (see section Motifs). (**D**) GERP (21) conservation scores of the gene of interest are plotted if available.

This section explains example output from AREsite2 for the gene Cxcl2 in *Homo sapiens*. If a search for the motifs ATTTA, WWTTTWW, GTTTG, TTTGTTT and AWTAAA is started, database entries are provided for the user as svg-plots and html5-tables. For visualization we use the R (R Core Team (2015)) package Gviz. The output begins with information on the genomic location of the searched gene. Figure 1A presents the ideogram of hg38 chromosome 4 with highlighted position of Cxcl2. Figure 1B visualizes the gene body and known transcripts of Cxcl2 as annotated by ENSEMBL. Annotated motifs, colored accordingly, if overlapping experimental data was available (see section Motifs) are highlighted in Figure 1C. All of these figures contain a link to the ENSEMBL genome browser, where selected motifs are made available as custom tracks. ENSEMBL (19) GERP (21) conservation scores for the whole gene body are visualized in Figure 1D where available.

**Figure 2.**
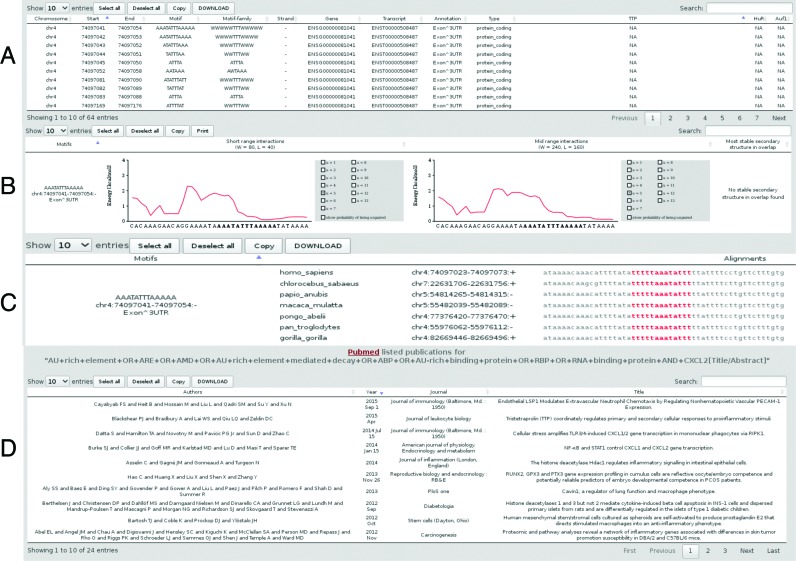
(**A**) Results table containing motifs of interest, their genic location and experimental evidence for RBP interaction if available. (**B**) Accessibility plot for a motif of interest, showing short- and mid-range basepair probabilities. The user has the option to investigate different settings of base pair distances (default 5nt). (**C**) Multiple sequence alignment of an annotated motif, the motif region is shown in red. (**D**) Results of the PUBMED literature search with via the ENTREZ API. The used search string is printed for an easy manual copy-and-paste literature search in the PUBMED interface.

The search for more sequence patterns and parsing of the whole gene body leads to an increase in predicted motifs. Table 2 shows a comparison of genes per genome containing at least one core ARE (AUUUA), GRE (GUUUG) and URE (UUUUU). To cope with this massive numbers and help users to filter potentially interesting candidates, we provide the second part of the results sections. The first table (Figure 2A) provides information on the genomic and genic location of an annotated motif, as well as experimental evidence for RBP interaction, if available. Accessibility or occupation of motifs by overlapping stable secondary structures, can be seen in the next table (Figure 2B). Detailed conservation information for each motif can be derived as multiple sequence alignment from table three (Figure 2C). Concluding table provides the results of the literature search, sorted by newest publications (Figure 2D). All tables are searchable, and content can be downloaded by the user.

**Table 2.** Genes with annotated A/U/GRE in AREsite2

Genome	Genes with ARE		Genes with URE		Genes with GRE	
	Exon	Intron	Exon	Intron	Exon	Intron
*H. sapiens*	31k	30k	24k	17k	24k	17k
*M. musculus*	24k	23k	18k	13k	18k	13k
*D. rerio*	17k	20k	10k	10k	11k	10k
*D. melanogaster*	13k	9k	8k	6k	8k	5k
*C. elegans*	19k	17k	16k	10k	13k	9k

Table 2 lists the number of genes with at least one ARE (AUUUA), GRE (GUUUG) and URE (UUUUU) in AREsite2 for all available genomes

## CONCLUSIONS AND PERSPECTIVES

AREsite2 presents a major update to AREsite, including three additional genomes and a high amount of newly annotated motifs. Furthermore, the new backend allows for easier integration of more genomes, other motifs, experimental and structure data. We provide the whole database as mysql-dump and all annotated motifs in bed, bed12 and gtf format for download. The RESTful service makes it easy for advanced users to retrieve information without the need to download any of these files in a semi-automatic manner. An example script for that purpose is included in the supplementary data, the most recent version can readily be downloaded from the website directly. We aim to integrate more experimental data as soon as they become available, either through CLIPdb, or directly from source if feasible.

## AVAILABILITY

The database is publicly available at: http://rna.tbi.univie.ac.at/AREsite

An example script for interaction with the REST interface, a database dump and motif annotation as bed, bed12 and gtf files are available at: http://rna.tbi.univie.ac.at/AREsite/bulk.

## SUPPLEMENTARY DATA

Supplementary Data are available at NAR online.
